# Breastfeeding and infant growth

**DOI:** 10.1093/emph/eov012

**Published:** 2015-07-01

**Authors:** Amber Gigi Hoi, Luseadra McKerracher

**Affiliations:** ^1^ Human Evolutionary Studies Program,; ^2^ Faculty of Health Sciences,; ^3^ Department of Archaeology, Simon Fraser University, 8888 University Dr., Burnaby, BC, Canada, V5A 1S6 and; ^4^ Centre for Biocultural History, Aarhus Universitet, Aarhus C, Denmark, 8000

## POOR INFANT GROWTH AND INSUFFICIENT BREASTMILK?

Globally, mothers from a wide variety of socio-environmental contexts often assume slow-growing babies are underfed and erroneously attribute perceived growth retardation to inadequate milk supply or poor milk quality [1]. These assumptions frequently prompt replacement of breastmilk with formula or other non-breastmilk foods to encourage infant weight and length gains [1]. This tendency to truncate breastfeeding to accelerate growth is exacerbated by some features of contemporary environments in both developing and developed nations such as growing rates of maternal obesity and caesarean section that interfere with breastfeeding.

Unfortunately, regardless of the primary reason for truncation, cessation of exclusive breastfeeding before 4–6 months and of continued breastfeeding before 12 months is associated with increased risk of gastrointestinal infections and poor immune system development in infancy, and obesity and a variety of non-communicable diseases in later life [2]. Although stunting and wasting in children *do* represent major public health challenges in low- and middle-income countries, these phenomena should not be confused with unfaltering growth less than two standard deviations below global averages.

## EVOLUTIONARY PERSPECTIVES

Abundant evidence shows breastfeeding surpasses formula feeding nutritionally, immunologically, and emotionally [2]. These findings are unsurprising, given that humans share with all other mammals > 200 million years of successful evolutionary history of milk production.

Within this broader mammalian context, humans have evolved both resilience and substantial flexibility in infant feeding. Regarding flexibility, use of complementary foods pre-weaning can partially offload the energetic burden of feeding from mothers to other caregivers [3]. Regarding resilience, nearly all human mothers can produce sufficient milk to meet infant needs—if not all demands—for the first 6 months postpartum even in adverse conditions, with milk production well-buffered against environmental insults [3]. Indeed, evidence from contemporary, non-industrialized populations indicates the most common infant feeding strategy is one of on-demand exclusive breastfeeding for ∼6 months, followed by introduction of easily digestible, nutrient-dense complementary foods combined with continued breastfeeding for 2–4 years. Our hunter-gatherer ancestors likely used infant feeding approaches similar to these norms, with variations attuned to local ecology [3].

Infant growth also likely varies with local population histories and environments. Growth trajectories vary widely within and especially among human populations, irrespective of infant feeding strategy [4].

## FUTURE IMPLICATIONS

Although new breastfeeding-based international infant growth standards (e.g. [5]) provide useful diagnostic guidelines regarding stunting, they should not be used to justify early cessation of exclusive or continued breastfeeding. Breastfeeding mothers can be assured that even very slow growth—if not interrupted by episodes of growth faltering—is often normal and healthy, only exceptionally rarely indicating milk insufficiency.

With the aim of reducing infectious and non-communicable disease burdens, clinicians should offer infant feeding advice that is both evidence-based and feasible within a given ecological, historical and social context. We should pay special attention to obese mothers and mothers that birthed via caesarean section, since these evolutionarily-novel factors negatively affect breastfeeding performance [6].

## FUNDING

AG receives support from Simon Fraser University (SFU). LM is supported two doctoral research fellowships, one provided by the Human Evolutionary Studies Program at SFU and one provided by the Social Sciences and Humanities Research Council of Canada, award # CGS-727-2011-33.
